# Impact of Mitochondrial DNA Mutations on Carotid Intima-Media Thickness in the Novosibirsk Region

**DOI:** 10.3390/life10090160

**Published:** 2020-08-22

**Authors:** Tatiana V. Kirichenko, Anastasia I. Ryzhkova, Vasily V. Sinyov, Marina D. Sazonova, Varvara A. Orekhova, Vasily P. Karagodin, Elena V. Gerasimova, Mikhail I. Voevoda, Alexander N. Orekhov, Yulia I. Ragino, Igor A. Sobenin, Margarita A. Sazonova

**Affiliations:** 1 Laboratory of Cellular and Molecular Pathology of Cardiovascular System, Research Institute of Human Morphology, 3 Tsyurupy Str., 117418 Moscow, Russia; a.h.opexob@gmail.com (A.N.O.); igor.sobenin@gmail.com (I.A.S.); 2 Laboratory of Angiopathology, Institute of General Pathology and Pathophysiology, 8 Baltiyskaya Str., 125315 Moscow, Russia; ryzhkovaai@gmail.com (A.I.R.); centaureaceanus@mail.ru (V.V.S.); marinasazon1990@gmail.com (M.D.S.); varvaraao@gmail.com (V.A.O.); vpka@mail.ru (V.P.K.); daisy29@mail.ru (M.A.S.); 3 Laboratory of Medical Genetics, National Medical Research Center of Cardiology, 15A 3 Cherepkovskaya Str., 121552 Moscow, Russia; 4 Institute for Atherosclerosis Research, Skolkovo Innovative Center, 143025 Moscow, Russia; 5 Department of Commodity Science and Expertise, Plekhanov Russian University of Economics, 125993 Moscow, Russia; 6 V.A.Nasonova Research Institute of Rheumatology, 34A Kashirskoe sh., 115522 Moscow, Russia; gerasimovaev@list.ru; 7 Research Institute of Internal and Preventive Medicine–Branch of the Institute of Cytology and Genetics, Siberian Branch of Russian Academy of Sciences, 630089 Novosibirsk, Russia; mvoevoda@ya.ru (M.I.V.); ragino@mail.ru (Y.I.R.)

**Keywords:** atherosclerosis, carotid intima-media thickness, mitochondrial mutations, cardiovascular risk factors

## Abstract

The search for markers of predisposition to atherosclerosis development is very important for early identification of individuals with a high risk of cardiovascular disease. The aim of the present study was to investigate the association of mitochondrial DNA mutations with carotid intima-media thickness and to determine the impact of mitochondrial heteroplasmy measurements in the prognosis of atherosclerosis development. This cross-sectional, population-based study was conducted in 468 subjects from the Novosibirsk region. It was shown that the mean (carotid intima-media thickness) cIMT correlated with the following mtDNA mutations: m.15059G>A (*r* = 0.159, *p* = 0.001), m.12315G>A (*r* = 0.119; *p* = 0.011), m.5178C>A (*r* = 0.114, *p* = 0.014), and m.3256C>T (*r* = 0.130, *p* = 0.011); a negative correlation with mtDNA mutations m.14846G>A (*r* = −0.111, *p* = 0.042) and m.13513G>A (*r* = −0.133, *p* = 0.004) was observed. In the linear regression analysis, the addition of the set of mtDNA mutations to the conventional cardiovascular risk factors increased the ability to predict the cIMT variability from 17 to 27%. Multi-step linear regression analysis revealed the most important predictors of mean cIMT variability: age, systolic blood pressure, blood levels of total cholesterol, LDL and triglycerides, as well as the mtDNA mutations m.13513G>A, m.15059G>A, m.12315G>A, and m.3256C>T. Thus, a high predictive value of mtDNA mutations for cIMT variability was demonstrated. The association of mutation m.13513G>A and m.14846G>A with a low value of cIMT, demonstrated in several studies, represents a potential for the development of anti-atherosclerotic gene therapy.

## 1. Introduction

Current knowledge about the mechanisms of atherosclerosis development has significantly increased in recent decades, but atherosclerotic-based diseases still occupy a leading position in the structure of mortality in developed countries [1]. In this regard, the early detection and treatment of patients with a high risk of atherosclerosis development is a primary medical and social problem. Timely prevention of subclinical atherosclerosis has a potential to decrease cardiovascular morbidity and mortality. The search for markers of subclinical atherosclerosis is one of the fundamental factors in the identification of individuals with a high risk of cardiovascular disease (CVD). The thickness of the intimal-medial layer of the carotid arteries (cIMT) is considered to be a direct non-invasive marker of subclinical atherosclerosis, used in clinical and epidemiological studies to assess the effect of conventional and new cardiovascular risk factors (CVRFs) on the progression of atherosclerotic lesions [2,3]. However, conventional CVRFs are poorly associated with cIMT, which suggests the presence of other factors determining the risk atherosclerosis development [4,5].

Currently, the existence of a genetic predisposition to the development of atherosclerosis is not in doubt. In particular, a family history of CVD (coronary artery disease, acute myocardial infarction, and arterial hypertension in first-degree relatives under 60 years of age) is an independent predictor of the risk of these diseases and is taken into account in many modern algorithms for cardiovascular risk prediction [6]. The association of numerous polymorphisms of the nuclear genome with risk of CVD has been revealed [7]. At the same time, the effect of mutations of the mitochondrial genome is more significant for the formation of atherosclerotic lesions since mitochondrial dysfunction leads to activation of the key factors of atherogenesis, such as oxidative stress and inflammation [8]. Nowadays, the role of mitochondrial genome mutations in the predisposition to atherosclerosis development and cardiovascular disease is being investigated [9].

In our previous studies, variants of mitochondrial heteroplasmy were identified in samples of the human aorta with atherosclerotic lesions [10]. In several populations, the association of mitochondrial mutations in blood leukocytes with coronary artery disease and carotid atherosclerosis has been studied [11,12,13]. It was revealed in the IMPROVE study, a European multicenter study aimed to investigate the prognostic value of cIMT for future cardiovascular events, that latitude is an important determinant of cIMT in Europe and traditional CVRFs do not fully explain cIMT variability in European population, which suggests the presence of other factors, including hereditary ones, in formation of a predisposition to atherosclerosis development [14]. In this regard, replicative studies on the association of mitochondrial heteroplasmy and carotid atherosclerosis need to be carried out in other regions that differ in ethnic composition, as well as in socio-economic and geographical conditions. We have previously investigated the association between mitochondrial heteroplasmy and carotid atherosclerosis in a population of the European part of Russia [15]. The objective of the present research is to study the possible relationship of mitochondrial heteroplasmy and cIMT variability in the Siberian region.

## 2. Results

In total, 500 participants were included in the study; 32 of them were excluded from the analysis due to unreadable ultrasound images (13) and the insufficient quality of their DNA samples (19). Therefore, 134 male and 334 female samples were analyzed. The clinical and laboratory characteristics of the study participants are presented in Table 1. Female and male groups were different by the following parameters: there were significantly more smokers in the male group (*p* = 0.038); men had higher systolic blood pressure (*p* = 0.001) and a lower level of high-density lipoproteins (*p* = 0.032). The additional estimated parameter in the female group was the period after menopause, which averaged 12.4 (7.4) years.

**Figure 1:**
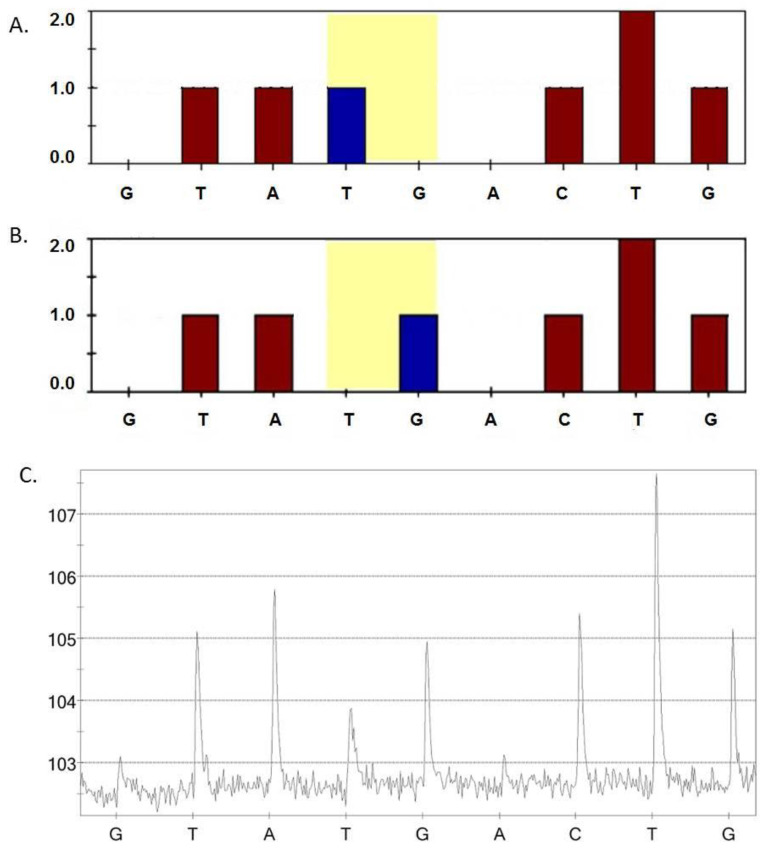
Sample of the sequencing results for mutation m.5178C>A. For the detection of mutation m.5178C>A, a reverse primer for sequencing was used, so the replacement of G by T was analyzed. (**A**) The theoretical height of the nucleotide peaks in homoplasmy for the mutant allele 5178A in the mitochondrial genome; (**B**) the theoretical height of the nucleotide peaks in homoplasmy for the normal allele 5178C in the mitochondrial genome; and (**C**) a pyrogram of a study participant’s DNA sample revealed the heteroplasmy level for mutation m.5178C>A is 48%.

Mitochondrial DNA (mtDNA) mutations m.13513G>A, m.3336T>C, m.15059G>A, m.12315G>A, m.1555A>G, m.5178C>A, m.14459G>A, m.14846G>A, and m.3256C>T were estimated in all the study participants. Figure 1 represents an example of the results of the pyrosequencing for mutation m.5178.

Table 2 demonstrates the levels of mtDNA mutations in total and in men and women separately. The difference between the male and female group was observed only for mutation m.15059G>A, which was significantly higher in women (*p* = 0.009).

Further, the association of mtDNA with mean cIMT was estimated and demonstrated in Table 3. In the total group, the mean cIMT correlated significantly with the following mtDNA mutations: m.15059G>A, m.12315G>A, m.5178C>A, and m.3256C>T, as well as inversely correlated with m.13513G>A and m.14846G>A. While the total group was divided by sex, the negative correlation of m.13513G>A with mean cIMT was observed in both men and women, while the correlation of m.14846G>A with cIMT reached statistical significance neither in men nor in women. A positive correlation of mutations m.15059G>A, m.12315G>A, and m.5178C>A was found only in women and m.3256C>T only in men.

The next stage of the study was a multi-step regression analysis that was performed to identify the factors that may affect cIMT progression in a population from the Novosibirsk region. Mean cIMT was used as a dependent variable, while variants of mitochondrial heteroplasmy as well as conventional cardiovascular risk factors were used as independent variables. The initial independent variables were age, sex, systolic and diastolic blood pressure, BMI, status of smoking, diabetes, a family history of CVD, parameters of the lipid profile—total cholesterol, triglycerides, and low- and high-density lipoproteins—as well as the levels of all the determined mtDNA mutations. The initial linear regression model, including the 20 variables listed above, explained the variability of mean cIMT with *r* = 0.521, *r*^2^ = 0.274, *p* < 0.001. The least significant independent variables were sequentially excluded from the analysis to obtain the most predictive linear regression model. In the resulting linear regression model (*r* = 0.462, *r*^2^ = 0.218, *p* < 0.001) the following parameters determined the variability of the dependent variable, mean cIMT: age, systolic blood pressure, blood levels of total cholesterol, LDL and triglycerides, and the mtDNA mutations m.13513G>A, m.15059G>A, m.12315G>A, and m.3256C>T. The exclusion of all mtDNA mutations from the initial analysis led to a decrease in the determination coefficient, i.e., deteriorate the explanatory capacity of the model to *r* = 0.413, *r*^2^ = 0.171, *p* < 0.001. A set of levels of mitochondrial mutations without other factors explained the variability of mean cIMT with *r* = 0.333, *r*^2^ = 0.111, *p* < 0.001.

In women, the initial set of predictors determined a cIMT value with *r* = 0.583, *r*^2^ = 0.340, *p* = 0.005; the most predictive variables were age, period after menopause, diabetes, blood levels of total cholesterol, LDL and triglycerides, and mtDNA mutations m.13513G>A, m.14459G>A, and m.5178C>A (*r* = 0.540, *r*^2^ = 0.292, *p* < 0.001). For men, the initial linear regression model was not significant (*r* = 0.657, *r*^2^ = 0.431, *p* = 0.227); the most important predictors of mean cIMT variability in the male group were age, blood levels of total cholesterol, HDL and triglycerides, smoking, diabetes, and mitochondrial heteroplasmy m.3256C>T (*r* = 0.600, *r*^2^ = 0.360, *p* = 0.003).

## 3. Discussion

In present study, the relationship between mutations of the mitochondrial genome with carotid atherosclerosis in the Novosibirsk region was studied for the first time. It was demonstrated that variants of mitochondrial heteroplasmy m.15059G>A, m.12315G>A, m.5178C>A, and m.3256C>T significantly correlated with the intima-media thickness of common carotid arteries; a negative correlation of m.13513G>A and m.14846G>A with mean cIMT was revealed. When a correlation analysis was carried out in men and women separately, it was shown that the mean cIMT correlated with m.13513G>A, m.15059G>A, m.12315G>A, m.5178C>A, and m.14459G>A in the female group and only with m.13513G>A and m.3256C>T in the male group. Probably, these results are associated with one of the main limitations in this study—the unequal volume of the male and female groups, so most correlations did not reach statistical significance in male group due to the small sample size. In previous studies, the association of variants of mitochondrial heteroplasmy was demonstrated in other populations. The association of mitochondrial heteroplasmy in the European part of Russia partially coincides with the results of the present study in the Siberian region. A previous study in the Moscow region revealed the positive correlation between carotid atherosclerosis and the following mitochondrial mutations: m.652delG, m.3256C>T, m.3336T>C, m.5178C>A, m.12315G>A, m.14459G>A, and m.15059G>A; and an inverse correlation for m.652insG, m.13513G>A, and m.14846G>A. [15]. In subjects derived from a Kazakhstan population, m.12315G>A was associated with cIMT only in women, but a negative correlation of m.13513G>A with cIMT was confirmed in the total group [16].

The present study also included an estimation of the predictive value of mitochondrial heteroplasmy for cIMT variability along with conventional CVRFs using a linear regression analysis. The linear regression model, including a set of conventional cardiovascular risk factors together with the investigated mitochondrial mutations, predicted the cIMT variability with *r*^2^ = 0.274. The coefficient of determination *r*^2^ shows that this set of factors explains only 27% of variability of the mean cIMT in the presented linear regression model; however, the exclusion of the levels of mitochondrial heteroplasmy from the analysis led to a decrease in the predictive ability of this model by 10% (*r*^2^ = 0.171), which indicates a high contribution of mitochondrial mutations in cIMT variability in participants of the present study. Exclusion of the non-significant variables during the multi-step regression analysis resulted in a decrease in the determination coefficient to 0.218, i.e., reduced the explanting ability of the model to 22%, but allowed identifying the most predictive variables—age, systolic blood pressure, parameters of the lipid profile, and the mtDNA mutations m.13513G>A, m.15059G>A, m.12315G>A, m.3256C>T. In our previous study, the factors affecting the cIMT value in subjects from Moscow were determined. It was shown that the same set of conventional cardiovascular risk factors explained 21% of the cIMT variability. The inclusion of the m.652delG, m.3256C>T, m.13513G>A, m.14459G>A, and m.15059G>A heteroplasmy levels in the linear regression model provided a significantly better explanatory level of 36% [17].

The association of mitochondrial genome mutations, occurring during ontogenesis or inherited through the maternal line, with the development of a number of pathological conditions in humans is widely known [18,19]. One more limitation of the present study was the fact that detected levels of mitochondrial heteroplasmy did not reach 50%, while it is known that the level of mitochondrial heteroplasmy should exceed 50% for development of clinical manifestations [20]. However, a number of other studies have demonstrated a significant relationship between cardiovascular diseases associated with atherosclerosis and some mutations in the mitochondrial genome, determined at a level comparable to the results of the current study. For example, in a study of 482 patients with coronary heart disease, the variant of mitochondrial heteroplasmy m.16189T>C, at a level of 21.6%, was associated with an increased risk of coronary heart disease compared to healthy participants who had a 4.5% prevalence of m.16189T>C [21]. In another study it was shown that mtDNA with a 4977 bp deletion in blood cells was at significantly higher level in patients with coronary artery disease in comparison with healthy subjects (26.2% vs. 4.5%) [22].

## 4. Materials and Methods

### 4.1. Study Design

The present study aimed to investigate the association of mitochondrial mutations with cIMT and to determine the most important factors affecting the development of carotid atherosclerosis in a Siberian population with a special focus on genetic predisposition, namely, mtDNA mutations. This cross-sectional, population-based trial was conducted in men and women aged 55–79 years from the Novosibirsk region. Conventional CVRFs, such as body mass index (BMI), systolic and diastolic blood pressure, smoking status, history diabetes mellitus, family history of CVD, treatment of arterial hypertension, blood lipids parameters (total cholesterol, triglycerides, and low- and high-density lipoproteins) were assessed. All study participants were free of CVD. The recruitment of patients was carried out at the Research Institute of Internal and Preventive Medicine, Novosibirsk, Russia. The additional inclusion criterion in the female group was time after menopause (>5 years). History of angina pectoris, myocardial infarction, intermittent claudication, stroke or transient ischemic attacks, as well as arterial revascularization was exclusion criteria. The thickness of the intima-media layer of the common carotid arteries was determined as a direct quantitative characteristic of carotid atherosclerosis. Blood samples of the study participants were obtained to measure the lipid profile and the levels of mtDNA mutations previously identified as related to atherosclerosis and cardiovascular disease. The study was performed in accordance with the Declaration of Helsinki of 1975 and its revised version of 2013. The study protocol was approved by the Institute for Atherosclerosis Research Committee on Human Research (Moscow, Russia). All study participants provided written informed consent prior the inclusion in the study.

### 4.2. Measurements of cIMT

B-mode high-resolution ultrasonography with a linear array vascular probe 7.5 MHz on ultrasonic scanner SonoScape SSI-1000 (SonoScape, Shenzhen, China) was used for examination of the carotid arteries. The left and right common carotid arteries, the carotid bifurcation area, as well as the external and internal carotid arteries were scanned [14]. The measurements of cIMT were performed at the far wall of the common carotid artery 10 mm opposite the top of the carotid bifurcation in three fixed projections—lateral, anterolateral, and posterolateral. The carotid ultrasound was carried out by one researcher throughout the study. Frozen images of the far wall of right and left carotid arteries in three projections (6 images for each person) were saved for subsequent analysis using the dedicated software package M’Ath (Metris, SRL, Argenteuil, France). The cIMT was measured as the distance from the leading edge of the first echogenic area to the leading edge of the second echogenic area. The average of six measurements was considered as an integral indicator of the intima-media thickness.

### 4.3. DNA Isolation, PCR and Pyrosequencing

To perform pyrosequencing, total mtDNA was isolated from whole venous blood by phenol-chloroform extraction using the method previously developed by our group on the basis of the method by Maniatis et al. [23]. Polymerase chain reaction (PCR) was performed to obtain fragments of mtDNA containing the region of the investigated mutations [24]. MtDNA samples were kept in TE buffer at a concentration of 0.03 μg/μL. The analysis of the heteroplasmy level in the mutations was carried out using the quantitative method, developed by M. A. Sazonova et al. Pyrosequencing was performed by using the PSQ HS96MA (Biotage, Uppsala, Sweden) device, to determine the defective allele quantified by analyzing the peak heights in the pyrogram of the one-chained PCR-fragments of a mitochondrial genome [25]. The heteroplasmy level was measured as a percent of the mtDNA mutant copies. In our previous study we investigated 40 mitochondrial mutations in samples of the aorta’s intima and revealed a high level of heteroplasmy for some mutations in the atherosclerotic lesions [24]. Further, these mutations were selected to assess the relationship between mitochondrial heteroplasmy in the blood leukocytes and ultrasound indicators of carotid atherosclerosis. The levels of the following mtDNA mutations were measured: m.13513G>A, m.3336T>C, m.15059G>A, m.12315G>A, m.1555A>G, m.5178C>A, m.14459G>A, m.14846G>A, and m.3256C>T.

### 4.4. Statistical Analysis

Statistical analysis was performed using SPSS 27.0 (IBM SPSS Statistics, IBM Corp., Armonk, NY, USA,) [26]. A Kolmogorov–Smirnov test with Lilliefors’s correction was performed to estimate the data distribution. The comparison of mean values for continuous variables was performed by a Mann–Whitney test, and for categorical variables by a chi-square Pearson’s test. Results were expressed in terms of the mean and standard deviation. Significance was defined at the 0.05 level of confidence. Pearson’s correlation coefficient was used for the correlation analysis between the cIMT and mtDNA mutations. Multi-step linear regression analysis was performed to identify the factors affecting the cIMT.

## 5. Conclusions

Association of the mtDNA mutations m.12315G>A, m.15059G>A, m.3256C>T, and m.5178C>A, and an inverse correlation of m.13513G>A and m.14846G>A, with thickening of the intimal-medial layer of carotid arteries was found in the study participants from the Novosibirsk region. The set of investigated mtDNA mutations had a high predictive value for cIMT variability and increased the explanatory ability of the linear regression models by 10% when added to conventional cardiovascular risk factors. The strong association of the mutation m.13513G>A and m.14846G>A with a low value of cIMT, demonstrated in several studies, represents a potential for the development of anti-atherosclerotic gene therapy, but further replication studies in other populations and in larger samples are required.

## Figures and Tables

**Table 1:** Clinical and laboratory characteristics of the study participants.

Characteristic	Total Group	Men	Women	Difference, *p*-Value
Age, years	62.3 (5.7)	62.9 (5.4)	62.1 (5.8)	0.141
BMI, kg/m^2^	28.6 (4.9)	28.4 (4.9)	28.7 (4.9)	0.515
SBP, mm Hg	130 (16)	134 (18)	127 (15)	0.001
DBP, mm Hg	81 (10)	82 (9)	80 (10)	0.121
Smoking, %	9	14	7	0.038
Diabetes, %	6	3	8	0.190
Family history of CVD, %	34	32	35	0.398
Hypotensive therapy, %	55	51	57	0.442
Total Cholesterol, mg/dL	231.7 (42.3)	227.4 (40.4)	233.5 (42.9)	0.131
TG, mg/dL	119.1 (73.3)	120.9 (74.6)	118.4 (72.8)	0.732
HDL, mg/dL	49.8 (14.2)	47.8 (12.8)	50.6 (14.7)	0.032
LDL, mg/dL	158.5 (38.3)	155.2 (37.0)	159.8 (38.8)	0.213
cIMT, mm	0.778 (0.170)	0.772 (0.149)	0.777 (0.150)	0.731

**Table 2:** The MtDNA mutation levels of the study participants.

MtDNA Mutations, %	Total Group	Men	Women	Difference, *p*-Value
m.13513G>A	24.3 (13.8)	23.8 (13.5)	24.5 (13.9)	0.635
m.3336T>C	3.9 (0.4)	3.1 (4.7)	4.2 (9.5)	0.086
m.15059G>A	11.7 (14.3)	9.5 (9.3)	12.6 (15.8)	0.009
m.12315G>A	36.0 (24.1)	35.4 (23.3)	36.2 (24.4)	0.714
m.1555A>G	22.8 (12.5)	24.2 (12.8)	22.2 (12.4)	0.070
m.5178C>A	5.8 (12.3)	4.8 (11.3)	6.3 (12.7)	0.216
m.14459G>A	4.1 (5.5)	4.4 (5.4)	4.1 (5.6)	0.540
m.14846G>A	17.1 (16.6)	17.7 (18.5)	16.9 (15.8)	0.654
m.3256C>T	15.0 (16.3)	15.4 (15.5)	14.9 (16.7)	0.772

**Table 3:** Correlation of mtDNA mutations with mean cIMT.

MtDNA Mutations (*r*; *p*)	Total Group	Men	Women
m.13513G>A	−0.133 *; 0.004	−0.173 *; 0.048	−0.119 *; 0.030
m.3336T>C	0.064; 0.176	−0.101; 0.251	0.097; 0.079
m.15059G>A	0.159 *; 0.001	0.006; 0.942	0.208 *; <0.001
m.12315G>A	0.119 *; 0.011	−0.040; 0.655	0.179 *; 0.001
m.1555A>G	−0.030; 0.525	−0.120; 0.193	0.011; 0.843
m.5178C>A	0.114 *; 0.014	−0.078; 0.375	0.181 *; 0.001
m.14459G>A	0.058; 0.220	−0.152; 0.085	0.138 *; 0.012
m.14846G>A	−0.111 *; 0.042	−0.144; 0.100	−0.071; 0.195
m.3256C>T	0.130 *; 0.011	0.297 *; 0.002	0.069; 0.257

*r*, Pearson’s correlation coefficient, with the significance of the correlation shown. *, statistical significance at *p* < 0.05.

## Abbreviations

BMI	Body mass index
cIMT	Carotid intima-media thickness
CVD	Cardiovascular disease
CVRFs	Conventional cardiovascular risk factors
DBP	Diastolic blood pressure
HDL	High-density lipoproteins
LDL	Low-density lipoproteins
mtDNA	Mitochondrial DNA
SBP	Systolic blood pressure

## References

[1] Benjamin E.J., Muntner P., Alonso A., Bittencourt M.S., Callaway C.W., Carson A.P., Chamberlain A.M., Chang A.R., Cheng S., Das S.R. (2019). Heart disease and stroke statistics-2019 update: A report from the American Heart Association. Circulation.

[2] Bots M.L., Evans G.W., Tegeler C.H., Meijer R. (2016). Carotid intima-media thickness measurements: Relations with atherosclerosis, risk of cardiovascular disease and application in randomized controlled trials. Chin. Med. J..

[3] Kirichenko T.V., Myasoedova V.A., Ravani A.L., Sobenin I.A., Orekhova V.A., Romanenko E.B., Poggio P., Wu W.-K., Orekhov A.N. (2020). Carotid atherosclerosis progression in postmenopausal women receiving a mixed phytoestrogen regimen: Plausible parallels with kronos early estrogen replacement study. Biology.

[4] Adams M.R., Nakagomi A., Keech A., Robinson J., McCredie R., Bailey B.P., Freedman S.B., Celermajer D.S. (1995). Carotid intima-media thickness is only weakly correlated with the extent and severity of coronary artery disease. Circulation.

[5] Lucaroni F., CicciarellaModica D., Macino M., Palombi L., Abbondanzieri A., Agosti G., Biondi G., Morciano L., Vinci A. (2019). Can risk be predicted? An umbrella systematic review of current risk prediction models for cardiovascular diseases, diabetes and hypertension. BMJ Open.

[6] Hindieh W., Pilote L., Cheema A., Al-Lawati H., Labos C., Dufresne L., Engert J.C., Thanassoulis G. (2016). Association between family history, a genetic risk score, and severity of coronary artery disease in patients with premature acute coronary syndromes. Arterioscler. Thromb. Vasc. Biol..

[7] Rankinen T., Sarzynski M.A., Ghosh S., Bouchard C. (2015). Are there genetic paths common to obesity, cardiovascular disease outcomes, and cardiovascular risk factors?. Circ. Res..

[8] Geto Z., Molla M.D., Challa F., Belay Y., Getahun T. (2020). Mitochondrial dynamic dysfunction as a main triggering factor for inflammation associated chronic non-communicable diseases. J. Inflamm. Res..

[9] Finsterer J. (2020). Atherosclerosis can be mitochondrial: A review. Cureus.

[10] Sobenin I.A., Sazonova M.A., Postnov A.Y., Bobryshev Y.V., Orekhov A.N. (2012). Mitochondrial mutations are associated with atherosclerotic lesions in the human aorta. Clin. Dev. Immunol..

[11] Kirichenko T.V., Sobenin I.A., Khasanova Z.B., Orekhova V.A., Melnichenko A.A., Demakova N.A., Grechko A.V., Orekhov A.N., Ble Castillo J.L., Shkurat T.P. (2018). Data on association of mitochondrial heteroplasmy and cardiovascular risk factors: Comparison of samples from Russian and Mexican populations. Data Brief.

[12] Mitrofanov K.Y., Zhelankin A.V., Shiganova G.M., Sazonova M.A., Bobryshev Y.V., Postnov A.Y., Sobenin I.A., Orekhov A.N. (2016). Analysis of mitochondrial DNA Heteroplasmic mutations A1555G, C3256T, T3336C, C5178A, G12315A, G13513A, G14459A, G14846A and G15059A in CHD patients with the history of myocardial infarction. Exp. Mol. Pathol..

[13] Sazonova M.A., Zhelankin A.V., Barinova V.A., Sinyov V.V., Khasanova Z.B., Postnov A.Y., Sobenin I.A., Bobryshev Y.V., Orekhov A.N. (2016). Dataset of mitochondrial genome variants associated with asymptomatic atherosclerosis. Data Brief.

[14] Baldassarre D., Nyyssönen K., Rauramaa R., de Faire U., Hamsten A., Smit A.J., Mannarino E., Humphries S.E., Giral P., Grossi E. (2010). Cross-sectional analysis of baseline data to identify the major determinants of carotid intima-media thickness in a European population: The IMPROVE study. Eur. Heart J..

[15] Sazonova M.A., Sinyov V.V., Ryzhkova A.I., Galitsyna E.V., Khasanova Z.B., Postnov A.Y., Yarygina E.I., Orekhov A.N., Sobenin I.A. (2017). Role of mitochondrial genome mutations in pathogenesis of carotid atherosclerosis. Oxid. Med. Cell. Longev..

[16] Kirichenko T.V., Ragino Y.I., Voevoda M.I., Urazalina S.J., Khasanova Z.B., Orekhova V.A., Sinyov V.V., Sazonova M.A., Orekhov A.N., Sobenin I.A. (2020). Data on association of mitochondrial heteroplasmy with carotid intima-media thickness in subjects from Russian and Kazakh populations. Data Brief.

[17] Sobenin I.A., Myasoedova V.A., Kirichenko T.V., Orekhova V.A., Khasanova Z.B., Sinyov V.V., Melnichenko A.A., Grechko A.V., Orekhov A.N. (2019). Profiling of risk of subclinical atherosclerosis: Possible interplay of genetic and environmental factors as the update of conventional approach. Vessel Plus.

[18] Wallace D.C. (2005). A mitochondrial paradigm of metabolic and degenerative diseases, aging, and cancer: A dawn for evolutionary medicine. Annu. Rev. Genet..

[19] Finsterer J. (2020). Secondary manifestations of mitochondrial disorders. J. Zhejiang Univ. Sci. B.

[20] Smith P.M., Lightowlers R.N. (2011). Altering the balance between healthy and mutated mitochondrial DNA. J. Inherit. Metab. Dis..

[21] Mueller E.E., Eder W., Ebner S., Schwaiger E., Santic D., Kreindl T., Stanger O., Paulweber B., Iglseder B., Oberkofler H. (2011). The mitochondrial T16189C polymorphism is associated with coronary artery disease in Middle European populations. PLoS ONE.

[22] Botto N., Berti S., Manfredi S., Al-Jabri A., Federici C., Clerico A., Ciofini E., Biagini A., Andreassi M.G. (2005). Detection of mtDNA with 4977 bp deletion in blood cells and atherosclerotic lesions of patients with coronary artery disease. Mutat. Res..

[23] Little P.F., Treisman R., Bierut L., Seed B., Maniatis T. (1983). Plasmid vectors for the rapid isolation and transcriptional analysis of human beta-globin gene alleles. Mol. Biol. Med..

[24] Sazonova M., Budnikov E., Khasanova Z., Sobenin I., Postnov A., Orekhov A. (2009). Studies of the human aortic intima by a direct quantitative assay of mutant alleles in the mitochondrial genome. Atherosclerosis.

[25] Sazonova M.A., Postnov A.I., Orekhov A.N., Sobenin I.A. (2011). A new method of quantitative estimation of mutant allele in mitochondrial genome. Patol. Fiziol. Èksperimental’naia Ter..

[26] IBM SPSS Statistics 27 Documentation. https://www.ibm.com/support/pages/downloading-ibm-spss-statistics-27/.

